# Empirical comparison of routinely collected electronic health record data for head and neck cancer‐specific survival in machine‐learnt prognostic models

**DOI:** 10.1002/hed.27241

**Published:** 2022-11-11

**Authors:** Damian P. Kotevski, Robert I. Smee, Claire M. Vajdic, Matthew Field

**Affiliations:** ^1^ Department of Radiation Oncology Prince of Wales Hospital and Community Health Services Sydney New South Wales Australia; ^2^ Prince of Wales Clinical School, Faculty of Medicine University of New South Wales Sydney New South Wales Australia; ^3^ Department of Radiation Oncology Tamworth Base Hospital Tamworth New South Wales Australia; ^4^ Centre for Big Data Research in Health, Faculty of Medicine University of New South Wales Sydney New South Wales Australia; ^5^ Kirby Institute, Faculty of Medicine University of New South Wales Sydney New South Wales Australia; ^6^ South Western Sydney Clinical School, Faculty of Medicine University of New South Wales Sydney New South Wales Australia; ^7^ Ingham Institute for Applied Medical Research Sydney New South Wales Australia

**Keywords:** cancer‐specific survival, head and neck cancer, machine learning, prediction models, prognostic factors

## Abstract

**Background:**

Knowledge of the prognostic factors and performance of machine learning predictive models for 2‐year cancer‐specific survival (CSS) is limited in the head and neck cancer (HNC) population.

**Methods:**

Data from our facilities' oncology information system (OIS) collected for routine practice (OIS dataset, *n* = 430 patients) and research purposes (research dataset, *n* = 529 patients) were extracted on adults diagnosed between 2000 and 2017 with squamous cell carcinoma of the head and neck.

**Results:**

Machine learning demonstrated excellent performance (area under the curve, AUC) in the whole cohort (AUC = 0.97, research dataset), larynx cohort (AUC = 0.98, both datasets), and oropharynx cohort (AUC = 0.99, both datasets). Tumor site and T classification were identified as predictors of 2‐year CSS in both datasets. Hypothyroidism and fitness for operation were further identified in the research dataset.

**Conclusions:**

Datasets extracted from an OIS for routine clinical practice and research purposes demonstrated high utility for informing 2‐year head and neck CSS.

## 1 INTRODUCTION

Cancer‐specific survival (CSS) is a critical oncological outcome measure used to understand the impact of cancer treatment.^1^, ^2^, ^3^ Machine learning (ML) prediction of 2‐year CSS in the head and neck cancer (HNC) population, at the site and subsite level, is relatively unexplored due to difficulties accessing death certificate data in hospital settings.

ML has many applications in radiation oncology, including outcome prediction, assisting with clinical decision‐making, treatment planning, image segmentation, and image guidance.^4^ Access to high‐quality and detailed health data in an unrestrictive and analytically supported format for ML analysis is a critical step to identify patterns in data, further the knowledge of disease prognosis, and guide clinicians to ultimately improve care and survival outcomes for patients with HNC. Cancer centers collect data in their oncology information systems (OIS) as part of routine clinical practice; however, OIS data quality and completeness for robust epidemiological HNC research remains questionable (Kotevski, 2022, unpublished). Therefore, it is unclear whether ML models developed using OIS data can reliably predict CSS outcomes.

Locoregional recurrence is a cancer‐specific outcome that occurs in up to 60% of patients within the first 2 years of diagnosis with HNC.^5^ These recurrences can be attributed to an increased risk of HNC‐related death within 2 years, especially if left untreated or deemed unsalvageable. CSS is another example of a cancer‐specific outcome, which provides an indication of the efficacy of treatment. Therefore, predicting 2‐year CSS may provide clinicians with a better understanding of treatment effects. Assessing a direct treatment decision aid based on a model is beyond the scope of this work; however, modeling treatment technique details combined with patient/disease characteristics and their association with CSS may lead to actionable treatment decision aids in the future.

To the best of our knowledge, there are no publicly available models which have predicted 2‐year CSS in HNC populations using clinical information, nor are there any available models whereby 2‐year CSS can be predicted for individual patients, since overall survival (OS) is the more commonly reported survival outcome in the HNC population. One study investigated 2‐year OS in a laryngeal carcinoma cohort using clinical information from trial and clinical cohorts, reporting poor and acceptable model performance, respectively.^6^ Other studies predicted OS at 5‐years with excellent performance in tongue^7^ and oral^8^ cancer cohorts, and at any time in an oral cancer cohort, reporting excellent performance.^9^

For the prediction of head and neck CSS, studies have developed models for oral cancer populations at 5‐years,^10^, ^11^, ^12^ and at 30‐months,^13^ with model performance ranging from excellent^10^ to acceptable,^11^ and poor.^13^ A model predicting CSS at any time in a hypopharyngeal cancer performed with acceptable performance.^14^

The aim of this study was to identify prognostic factors and develop and internally validate ML models for the prediction of 2‐year CSS in patients with HNC at the site and subsite level, using data collected at a major Australian metropolitan teaching and tertiary referral hospital.

## 2 MATERIALS AND METHODS

This study has been prepared in accordance with the TRIPOD statement for the transparent reporting of a multivariable prediction model for individual prognosis or diagnosis.^15^ For this study, type 2b prognostic analysis was performed.

### 2.1 Data sources

Content data in the OIS and research datasets for this study were sourced from the Prince of Wales Hospital Cancer Centre (POWCC) MOSAIQ oncology information system (OIS) as previously described by Kotevski et al. (unpublished). The OIS dataset contained routinely collected data for government reporting, extracted using structured query language. The research dataset contained manually curated data extracted from unstructured clinical documents and has been use in several publications.^1^, ^2^, ^3^, ^16^, ^17^, ^18^

Death data were sourced from the National Death Index (NDI) in February 2018 via probabilistic record linkage (EO2017/5/392), the NSW Registry of Births, Death and Marriages, hospital notifications, and the Ryerson Index (death notices and obituaries in Australian newspapers). NDI cause of death data were not available for patients registered as deceased in the 2 years prior to linkage (2016–2017).

### 2.2 Participants

Eligible patients were 18 years or older and presented between 1 January 2000 and 31 December 2017 with a newly diagnosed, previously untreated squamous cell carcinoma of the head and neck, including the hypopharynx, larynx, nasopharynx, oral cavity, oropharynx, paranasal sinus, and salivary glands. Further inclusion criteria were the receipt of definitive treatment with radiotherapy (with or without chemotherapy or surgery), known T and N classification, and nil distant metastasis at presentation. Patients were excluded if they presented for consultation only, received palliative treatment only, or presented with distant metastases. All patients were staged using the current edition Union for International Cancer Control TNM manual available at the time of diagnosis. The study was approved by the NSW Population and Health Services Research Ethics Committee (2019/ETH12196).

### 2.3 Outcome

The study outcome was 2‐year CSS where the event was death from HNC within 2 years of initial treatment. ICD‐10 cause of death codes were obtained through linkage with the NDI which the study investigators used to classify death as HNC or non‐HNC related.

### 2.4 Predictors

A total of 24 patient, tumor, and treatment available variables were assessed in multivariable Cox regression (CR) and ML prediction models (Table S1, Supporting Information). Human papillomavirus data were largely missing and was thus excluded from analysis. Figures S1 and S2 describe the overall study and ML analysis. The remaining 23 variables were assessed for missing data using Little's missing‐completely‐at‐random (MCAR) test in SPSS 27 (IBM, Armonk, NY). Variables with more than 20% MCAR were automatically excluded. Variables with less than 20% MCAR were imputed by modeling each variable containing missing data as a function of other variables in the dataset in ascending order (variables with fewest missing values to most) as a multivariate imputer (IterativeImputer).^19^ The imputation strategy was inspired by van Buuren et al.^20^ and utilized the most frequent value for each column, iterated 10 times. Imputed data were cross‐validated using the Repeated Stratified K‐Fold function with 3 repeats and 10 splits.

This process resulted in 23 predictors in the research dataset and 9 in the OIS dataset. Due to the heterogeneity and the differences in radiotherapy treatment protocols for each HNC site, a clinically significant categorical cut‐off point was not used to dichotomize radiotherapy dose, fraction, and treatment duration. These variables were dichotomized based on the median value for each variable. Age categorization was based on the median value for patients with HNC in NSW.^21^ The predictor variables fitness for operation and performance status are two different measures; for example, certain medical events (i.e., heart attack) can deem a patient unfit for operation yet they have a good performance status. Fitness for surgery is a measure that incorporates patient age, comorbidities, and functional capacity, while performance status is a measure of functional capacity alone. Inclusion of fitness for surgery is a more holistic measure of a patient's status which can potentially impact upon CSS.

### 2.5 Sample size

A total of 1105 patients presented with a diagnosis of HNC between 2000 and 2017. Of these, 529 were eligible in the research dataset, and 430 in the OIS dataset. Differences in population numbers were due to missing values in the variables constituting the inclusion criteria, predominantly TNM staging and treatment aim.

### 2.6 Statistical analysis

All predictors were reported as categorical variables; however, age and radiotherapy details were also examined as continuous variables. To identify prognostic factors for 2‐year CSS, univariable analysis was conducted for each variable in the OIS and research datasets using the Kaplan–Meier method. Statistical significance was determined using the log‐rank test. Variables statistically significant at the univariable level were included in a multivariable CR model with forward stepwise conditional selection. These factors were reported as hazard ratios (HR) alongside 95% confidence intervals (CI). For each variable, the reference category was the category with the highest frequency. The level of significance was *p* < 0.05 and all *p*‐values were two sided. Univariable and multivariable analysis was performed using SPSS 26.

Due to class imbalance in the distribution of the outcome variable in favor of survival in the OIS and research datasets, over‐sampling was performed using the imbalanced‐learn SMOTE (synthetic minority over‐sampling technique) Python package prior to splitting the dataset for cross‐validation and testing. The minority class (death due to HNC at 2 years) was over‐sampled by creating synthetic minority class samples along the line segments joining any minority class *k*‐nearest neighbors.^22^ Therefore, it is likely that each cross‐validation split and the test set contained a mixture of synthetic and real data.

To address potential bias in the OIS and research datasets, models were trained on three datasets, (1) the research dataset with 23 variables, (2) the OIS dataset with 9 variables, and (3) the research dataset restricted to the 9 variables in the OIS dataset. From these datasets, ML was applied to the whole cohort, and then independently on larynx, oral cavity, and oropharynx patients extracted from the whole cohort (Figure S1). Five ML classification models (logistic regression [LR], gradient boosted trees [GBT], random forest [RF], support vector machine [SVM], and artificial neural network [ANN]) were trained on 80% of the imputed and balanced dataset. Models were trained using fivefold cross‐validation and hyperparameter tuning in Python.^19^ All predictive features were used in the development of specific subsite models, with no feature selection performed per subsite. The models were internally tested on the remaining 20% of the dataset.

Models were compared on the commonly reported performance metrics; recall/sensitivity, specificity, precision, F1‐score, and receiver operating characteristic curve—area under the curve (AUC). The standard error for each metric was calculated from each model's respective confusion matrix and used to determine the 95% confidence interval.

## 3 RESULTS

Most patients were male, aged less than 65 years, with either an oropharynx, larynx, or oral cavity tumor, and almost half of the patients presented with local disease. Radiotherapy was delivered at a median dose of 65 gray, in a median of 32 fractions, over a median of 40 days. Almost a third (*n* = 161, 30%) of patients in the research dataset, and 69 (16%) patients in the OIS dataset were also treated with chemotherapy. Of note, chemotherapy data were only available from 2012 in the OIS dataset and complete in the research dataset. Almost half of the cohort (*n* = 212, 40%) in the research dataset were treated with surgery, while surgical data in the OIS dataset were missing as this information is not required for routine clinical reporting (Table S1). Six of the twelve patient variables (Table 1), six tumor variables (Table 2), and five treatment variables (Table 3) were assessed for association with 2‐year CSS.

**TABLE 1:** Patient variables influencing 2‐year head and neck cancer death in the whole cohort

Factor	Research dataset (*n* = 529)					OIS dataset (*n* = 430)				
	Frequency (%)	No. event (%)	2‐year CSS (SE)	Univariable *p*‐value	Multivariable HR (95% CI)^b^, *p*‐value	Frequency (%)	No. event (%)	2‐year CSS (SE)	Univariable *p*‐value	Multivariable HR (95% CI)^b^, *p*‐value
Age (years)^a^										
<65	314 (59%)	43 (14%)	85% (2%)	**0.029**	Ref	248 (58%)	31 (13%)	86% (2%)	0.056	
≥65	215 (41%)	44 (20%)	78% (3%)		0.55 (0.12–2.61), 0.459	182 (42%)	35 (19%)	79% (3%)		
Sex										
Male	432 (82%)	66 (15%)	84% (2%)	0.110		348 (81%)	53 (15%)	83% (2%)	0.827	
Female	97 (18%)	21 (22%)	77% (4%)			82 (19%)	13 (16%)	82% (5%)		
Hypothyroidism										
No	516 (98%)	82 (16%)	83% (2%)	**0.003**	Ref	NC	NC	NC	NC	
Yes	12 (2%)	5 (42%)	46% (17%)		**4.82 (1.86–12.44), 0.001**	NC	NC	NC		
Unknown	1 (<1%)	0								
Alcohol consumption										
Never	119 (22%)	24 (20%)	78% (4%)	**0.032**	1.07 (0.94–1.22), 0.300	NC	NC	NC	NC	
Social	137 (26%)	13 (10%)	90% (3%)		3.42 (0.92–12.64), 0.065	NC	NC	NC		
Daily	253 (48%)	45 (18%)	81% (3%)		Ref	NC	NC	NC		
Unknown	20 (4%)	0								
Fitness for operation										
No	33 (6%)	11 (33%)	59% (10%)	**<0.001**	**2.61 (1.27–5.38), 0.009**	NC	NC	NC	NC	
Yes	496 (94%)	76 (15%)	84% (2%)		Ref	NC	NC	NC		
Performance status										
0, normal	287 (54%)	34 (12%)	87% (2%)	**0.001**	Ref	35 (8%)	1 (3%)	96% (4%)	0.841	
1, symptoms/self‐care	204 (39%)	43 (21%)	78% (3%)		1.66 (0.77–3.58), 0.198	7 (2%)	0			
2, ambulatory <50%	15 (3%)	5 (33%)	63% (13%)		2.67 (0.82–8.68), 0.102	0	0			
3, ambulatory >50%	4 (1%)	2 (50%)	50% (25%)		0.15 (0–NE), 0.702	2 (1%)	0			
4, bedridden	1 (<1%)	0			NE	0	0			
Unknown	18 (3%)	0				386 (89%)	0			

*Note*: Unknown values were excluded from univariate and multivariate analysis. Bold indicates statistical significance.

Abbreviations: CI, confidence interval; CSS, cancer‐specific survival; HN, head and neck; HR, hazard ratio; NC, not collected; NE, not estimable; NS, not significant; OIS, oncology information system; Ref, reference category; SE, standard error.

^a^ Based on median value in NSW.^21^

^b^ 95% CI for variables with no statistically significant categories were manually calculated.^35^

**TABLE 2:** Tumor variables influencing 2‐year head and neck cancer death in the whole cohort

Factor	Research dataset (*n* = 529)					OIS dataset (*n* = 430)				
	Frequency (%)	No. event (%)	2‐year CSS (SE)	Univariable *p*‐value	Multivariable HR (95% CI)^b^, *p*‐value	Frequency (%)	No. event (%)	2‐year CSS (SE)	Univariable *p*‐value	Multivariable HR (95% CI)^b^, *p*‐value
Tumor site										
Hypopharynx	33 (6%)	9 (27%)	69% (9%)	**<0.001**	**2.50 (1.11–5.62), 0.027**	24 (6%)	7 (29%)	62% (12%)	**<0.001**	2.32 (0.98–5.48), 0.056
Larynx	153 (29%)	13 (9%)	91% (2%)		0.82 (0.42–1.62), 0.572	127 (29%)	7 (5%)	94% (2%)		0.49 (0.21–1.17), 0.110
Nasopharynx	23 (4%)	1 (4%)	96% (4%)		0.44 (0.06–3.35), 0.431	26 (6%)	2 (8%)	91% (6%)		0.65 (0.15–2.80), 0.560
Oral cavity	99 (19%)	36 (36%)	62% (5%)		**3.14 (1.82–5.44), <0.001**	86 (20%)	25 (29%)	65% (6%)		**3.25 (1.77–5.95), <0.001**
Oropharynx	209 (39%)	27 (13%)	86% (3%)		Ref	160 (37%)	21 (13%)	85% (3%)		Ref
Salivary glands	4 (1%)	1 (25%)	75% (22%)		1.92 (0.26–14.28), 0.523	7 (2%)	4 (57%)	43% (19%)		**6.79 (2.20–20.95), 0.001**
Paranasal sinus	8 (2%)	0			NE	0	0			NE
Tumor grade										
Well differentiated	43 (8%)	4 (9%)	90% (5%)	0.344		39 (9%)	5 (13%)	84% (7%)	0.212	
Moderately well differentiated	244 (46%)	45 (18%)	81% (3%)			124 (29%)	23 (19%)	80% (4%)		
Poorly differentiated	136 (26%)	20 (15%)	85% (3%)			67 (16%)	5 (8%)	92% (4%)		
Undifferentiated	0	0				4 (1%)	0			
Unknown	106 (20%)	0				196 (45%)	0			
Cancer operable										
No	60 (11%)	17 (28%)	70% (6%)	**0.006**	3.33 (0.91–12.14), 0.068	NC	NC	NC	NC	
Yes	465 (88%)	70 (15%)	84% (2%)		Ref	NC	NC	NC		
Unknown	4 (1%)									
T classification										
T1	165 (31%)	7 (4%)	96% (2%)	**<0.001**	Ref	136 (32%)	6 (4%)	95% (2%)	**<0.001**	Ref
T2	141 (27%)	23 (16%)	82% (3%)		**2.72 (1.14–6.49), 0.025**	121 (28%)	16 (13%)	85% (3%)		1.94 (0.74–5.08), 0.176
T3	153 (29%)	37 (24%)	74% (4%)		**5.95 (2.60–13.65), <0.001**	115 (27%)	30 (26%)	72% (4%)		**6.06 (2.49–14.75), <0.001**
T4	70 (13%)	20 (29%)	69% (6%)		**4.96 (1.96–12.55), 0.001**	58 (13%)	14 (24%)	67% (7%)		**4.89 (1.83–13.05), 0.002**
N classification										
N0	254 (48%)	40 (16%)	83% (2%)	0.916		207 (48%)	22 (11%)	87% (2%)	**0.040**	Ref
N1	101 (19%)	17 (17%)	82% (4%)			81 (19%)	16 (20%)	77% (5%)		0.01 (0‐NE), 0.914
N2	158 (30%)	28 (18%)	81% (3%)			132 (31%)	26 (20%)	78% (4%)		3.84 (1.00–14.76), 0.050
N3	16 (3%)	2 (13%)	86% (10%)			10 (2%)	2 (20%)	75% (16%)		1.45 (0.80–2.63), 0.229
TNM stage^a^										
I	93 (18%)	1 (1%)	99% (1%)	**<0.001**	EXC	84 (19%)	2 (2%)	98% (2%)	**0.002**	EXC
II	71 (13%)	17 (24%)	75% (5%)			54 (13%)	9 (17%)	82% (5%)		
III	145 (27%)	27 (19%)	80% (4%)			115 (27%)	23 (20%)	78% (4%)		
IV	220 (42%)	42 (19%)	79% (3%)			177 (41%)	32 (18%)	79% (3%)		

*Note*: Unknown values were excluded from univariate and multivariate analysis. Bold indicates statistical significance.

Abbreviations: CI, confidence interval; CSS, cancer‐specific survival; EXC, excluded; HR, hazard ratio; NC, not collected; NE, not estimable; NS, not significant; OIS, oncology information system; Ref, reference category; SE, standard error.

^a^ Due to high correlation with T and N classification, TNM stage was not included in the multivariate model.

^b^ 95% CI for variables with no statistically significant categories were manually calculated.^35^

**TABLE 3:** Treatment variables influencing 2‐year head and neck cancer death in the whole cohort

Factor	Research dataset (*n* = 529)					OIS dataset (*n* = 430)				
	Frequency (%)	No. event (%)	2‐year CSS (SE)	Univariable *p*‐value	Multivariable HR (95% CI)^a^, *p*‐value	Frequency (%)	No. event (%)	2‐year CSS (SE)	Univariable *p*‐value	Multivariable HR (95% CI)^a^, *p*‐value
Radiotherapy dose (Gy)										
<65	280 (53%)	48 (17%)	82% (2%)	0.879		208 (48%)	40 (19%)	79% (3%)	0.054	
≥65	249 (47%)	39 (16%)	83% (3%)			222 (52%)	26 (12%)	87% (3%)		
Radiotherapy fractions										
<32	269 (51%)	48 (18%)	81% (2%)	0.485		201 (47%)	37 (18%)	80% (3%)	0.100	
≥32	260 (49%)	39 (15%)	84% (2%)			229 (53%)	29 (13%)	86% (2%)		
Radiotherapy duration (days)										
<40	318 (60%)	55 (17%)	82% (2%)	0.717		189 (44%)	35 (19%)	80% (3%)	0.206	
≥40	211 (40%)	32 (15%)	84% (3%)			241 (56%)	31 (13%)	85% (2%)		
Chemotherapy										
No	368 (70%)	67 (18%)	81% (2%)	0.122		20 (5%)	2 (10%)	89% (7%)	0.154	
Yes	161 (30%)	20 (12%)	87% (3%)			69 (16%)	2 (3%)	97% (2%)		
Unknown	0	0				341 (79%)	0			
Surgery										
No	317 (60%)	41 (13%)	86% (2%)	**0.017**	Ref	NC	NC	NC	NC	
Yes	212 (40%)	46 (22%)	77% (3%)		0.08 (0‐NE), 0.779	NC	NC	NC		

*Note*: Unknown values were excluded from univariate and multivariate analysis. Bold indicates statistical significance.

Abbreviations: CI, confidence interval; CSS, cancer‐specific survival; Gy, gray; HR, hazard ratio; IQR, interquartile range; NC, not collected; NS, not significant; OIS, oncology information system; Ref, reference category; SE, standard error.

^a^ 95% CI for variables with no statistically significant categories were manually calculated.^35^

**FIGURE 1:**
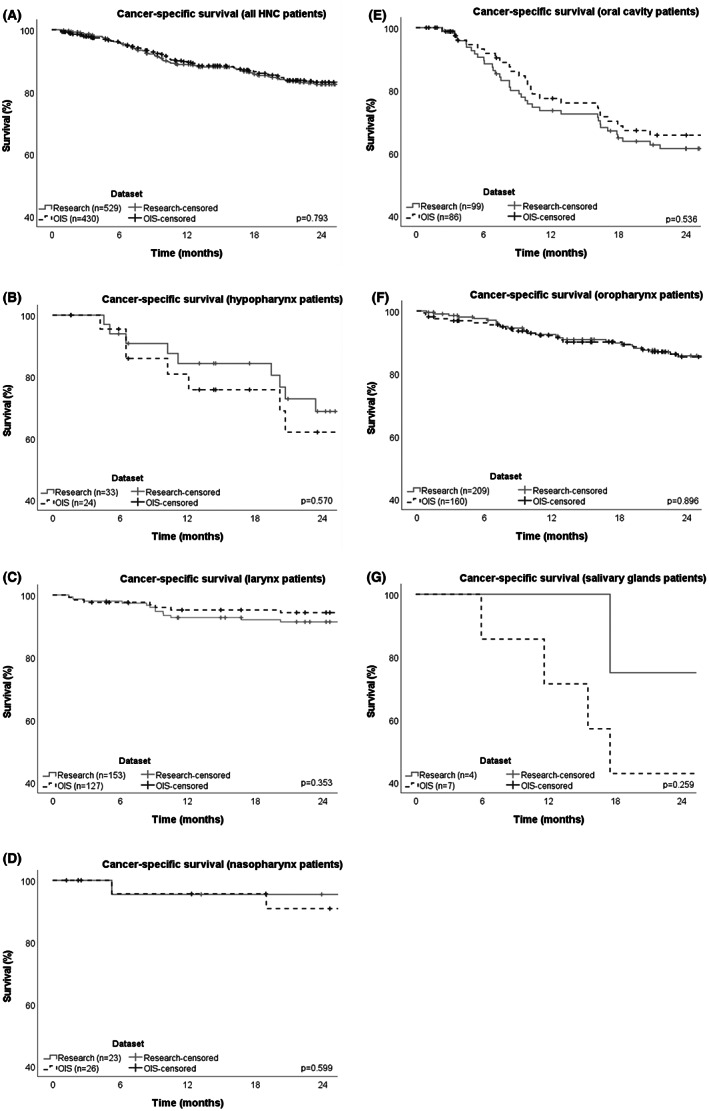
Kaplan–Meier 2‐year CSS by dataset, (A) whole cohort, (B) hypopharynx patients, (C) larynx patients, (D) nasopharynx patients, (E) oral cavity patients, (F) oropharynx patients, and (G) salivary glands patients. CSS, cancer‐specific survival; HNC, head and neck cancer; OIS, oncology information system

In the research dataset, a total of 87 (16%) deaths within 2 years were due to HNC, with a median CSS of 9.4 months and a 2‐year CSS rate of 83%. In the OIS dataset, 66 (15%) deaths within 2 years were due to HNC with a median CSS interval of 9.8 months and a 2‐year CSS rate of 83%. The 2‐year CSS rates by site for the OIS and research datasets, respectively, were hypopharynx 62% and 69%, larynx 95% and 91%, nasopharynx 91% and 96%, oral cavity 66% and 62%, oropharynx 85% and 86%, and salivary glands 43% and 75%. There was no statistical difference in 2‐year CSS between the two datasets at any site (Figure 1).

### 3.1 Research dataset prognostic factors

Five patient variables had a univariable association with 2‐year CSS (Table 1). Previous cancer diagnosis, diabetes, hypertension, and smoking status were also investigated but were not significant in univariable analyses (Table S1). Tumor site, whether a patient had operable cancer, T classification, and TNM stage were associated with 2‐year CSS at the univariable level (Table 2), while surgery was the only treatment variable associated with the outcome in univariable analyses (Table 3).

**FIGURE 2:**
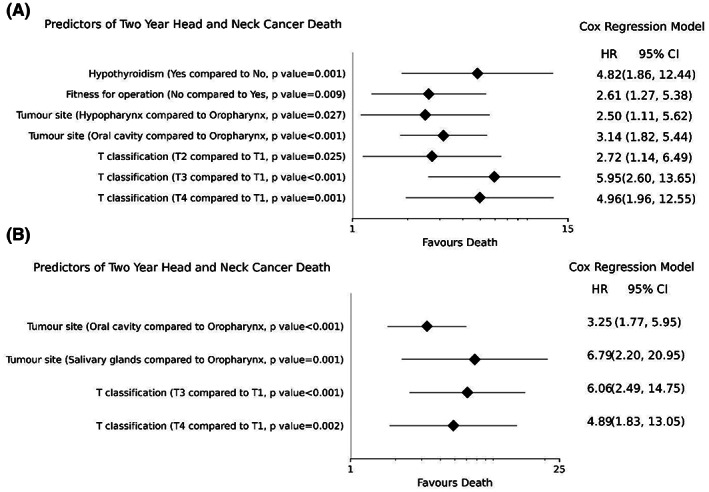
Forest plot of statistically significant predictors with multivariable association with 2‐year head and neck cancer death, (A) research dataset (23 variables analyzed) and, (B) OIS dataset (9 variables analyzed). HR, hazard ratio; OIS, oncology information system; 95% CI, 95% confidence interval

In multivariable analysis (Figure 2A), hypothyroidism (HR = 4.82, 95% CI 1.86–12.44, *p* = 0.001), lack of fitness for operation (HR = 2.61, 95% CI 1.27–5.38, *p* = 0.009), hypopharynx (HR = 2.50, 95% CI 1.11–5.62, *p* = 0.027) or oral cavity (HR = 3.14, 95% CI 1.82–5.44, *p* < 0.001) primary site compared to oropharynx, and T2 (HR = 2.72, 95% CI 1.14–6.49, *p* = 0.025), T3 (HR = 5.95, 95% CI 2.60–13.65, *p* < 0.001), or T4 (HR = 4.96, 95% CI 1.96–12.55, *p* = 0.001) compared to a T1 classification cancer were associated with increased risk of death from HNC. Due to high correlation between T, and N classification and TNM stage, the latter was excluded from the multivariable models.

### 3.2 Research dataset model validation

**FIGURE 3:**
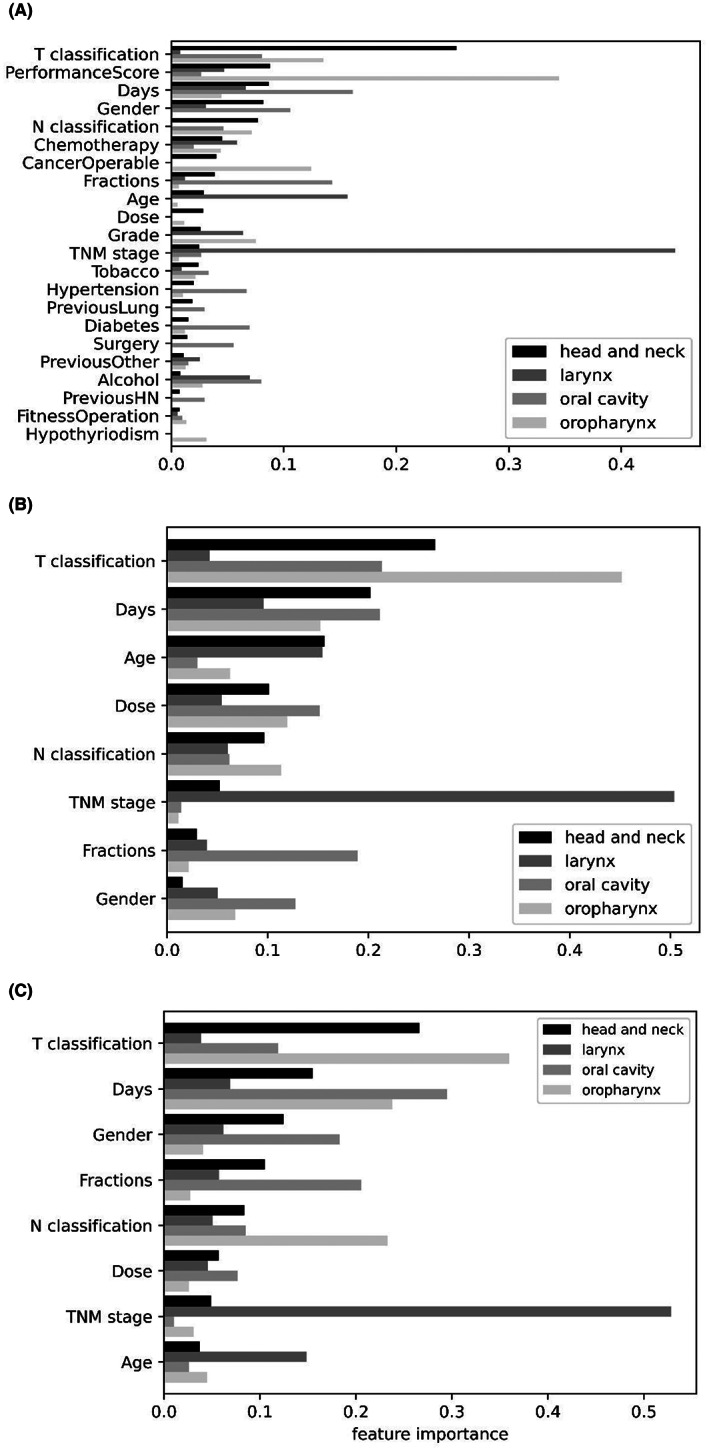
Decision tree feature importance for predicting 2‐year HNC death by site in the (A) research dataset, (B) OIS dataset, and (C) matched research dataset. OIS, oncology information system

The tree‐based models (GBT and RF) were consistently the highest performing models for each tumor site using the research dataset for the prediction of 2‐year CSS (Tables 4 and S2). In the whole cohort, the GBT and RF models had comparable performance, with the SVM model producing the highest recall. The GBT model performed the best in the larynx cohort; however, the LR, RF, and SVM models also demonstrated similarly high performance. No model performed well in the oral cavity cohort, reflective of the low sample size. In the oropharynx cohort, the RF model generated the highest metrics followed by the GBT model with slightly lower metrics. Models were not trained on the remaining tumor subsites due to the small sample sizes. Model feature importance for the prediction of 2‐year CSS is demonstrated in Figure 3.

**TABLE 4:** Summary of best machine learning model validation performance for each dataset

Site	Metric	Model performance metrics (95% CI)		
		Research dataset (*n* = 23 variables)	Clinical dataset (*n* = 9 variables)	Matched research dataset (*n* = 9 variables)
Whole cohort	Model	RF (GBT comparable)	RF	RF (GBT comparable)
	Recall	0.85 (0.78–0.92)	0.85 (0.77–0.93)	0.88 (0.81–0.95)
	Specificity	0.94 (0.89–0.99)	0.96 (0.92–1.00)	0.90 (0.84–0.96)
	Precision	0.94 (0.89–0.99)	0.95 (0.90–1.00)	0.90 (0.84–0.96)
	F1‐score	0.89 (0.84–0.94)	0.90 (0.85–0.95)	0.89 (0.84–0.94)
	AUC	0.97 (0.95–0.99)	0.94 (0.91–0.97)	0.95 (0.93–0.97)
Larynx	Model	GBT	RF	RF (GBT and SVM comparable)
	Recall	0.96 (0.89–1.00)	0.88 (0.75–1.00)	0.96 (0.89–1.00)
	Specificity	0.89 (0.77–1.00)	0.96 (0.88–1.00)	0.86 (0.73–0.99)
	Precision	0.90 (0.79–1.00)	0.95 (0.86–1.00)	0.87 (0.75–0.99)
	F1‐score	0.93 (0.86–1.00)	0.91 (0.83–0.99)	0.92 (0.85–0.99)
	AUC	0.98 (0.95–1.00)	0.98 (0.95–1.00)	0.97 (0.94–1.00)
Oral cavity	Model	RF	GBT	SVM
	Recall	0.62 (0.36–0.88)	0.67 (0.40–0.94)	0.77 (0.54–1.00)
	Specificity	0.69 (0.44–0.94)	0.69 (0.44–0.94)	0.62 (0.36–0.88)
	Precision	0.67 (0.40–0.94)	0.67 (0.40–0.94)	0.67 (0.43–0.91)
	F1‐score	0.64 (0.45–0.83)	0.67 (0.48–0.86)	0.71 (0.54–0.88)
	AUC	0.69 (0.55–0.83)	0.72 (0.58–0.86)	0.67 (0.52–0.82)
Oropharynx	Model	RF	RF (GBT comparable)	GBT (RF comparable)
	Recall	0.92 (0.83–1.00)	0.89 (0.77–1.00)	0.89 (0.79–0.99)
	Specificity	0.97 (0.92–1.00)	0.96 (0.89–1.00)	0.92 (0.83–1.00)
	Precision	0.97 (0.91–1.00)	0.96 (0.88–1.00)	0.91 (0.82–1.00)
	F1‐score	0.94 (0.88–1.00)	0.93 (0.86–1.00)	0.90 (0.83–0.97)
	AUC	0.99 (0.97–1.00)	0.98 (0.95–1.00)	0.93 (0.87–0.97)

Abbreviations: AUC, area under curve; GBT, gradient boosted tree; RF, random forest; SVM, support vector machine; 95% CI, 95% confidence interval.

### 3.3 Oncology information system dataset prognostic factors

None of the patient variables in the OIS dataset were associated with 2‐year CSS in univariable analyses (Table 1). Within the tumor variables, tumor site, T classification, N classification, and TNM stage were statistically significant in univariable analyses (Table 2), while all treatment variables were insignificant (Table 3). In multivariable analysis, compared to patients with oropharynx tumors, patients with oral cavity (HR = 3.25, 95% CI 1.77–5.95, *p* < 0.001) and salivary gland tumors (HR = 6.79, 95% CI 2.20–20.95, *p* = 0.001) were more likely to die from HNC within 2 years (Figure 2B). Patients with hypopharyngeal primary site demonstrated a trend for worse CSS (HR = 2.32, 95% CI 0.98–5.48, *p* = 0.056) compared to those with an oropharyngeal primary site. A higher T classification was associated with worse CSS, where patients with T3 classification (HR = 6.06, 95% CI 2.49–14.75, *p* < 0.001) and T4 classification (HR = 4.89, 95% CI 1.83–13.05, *p* = 0.002) were more likely to die from HNC within 2 years compared to patients with T1 classification. N classification was not significant in the multivariable model.

### 3.4 Oncology information system dataset model validation

The availability of fewer predictors did not significantly reduce the performance of the models. The tree‐based models (GBT and RF) yielded high metrics for the prediction of 2‐year CSS using the OIS dataset (Tables 4 and S3). In the whole HNC and larynx cohorts, the RF model best predicted 2‐year CSS, with comparable performance by the SVM model in the larynx cohort. Consistent with the research dataset, prediction of 2‐year CSS in the oral cavity cohort was poor with no suitable model. The GBT and RF models generated the highest metrics in the oropharynx cohort, with the ANN model scoring perfect specificity and precision but poor recall. Stage was the most influential feature in the prediction of 2‐year CSS (Figure 3). To highlight the importance of T classification in multivariable ML analysis, univariable analysis with T classification and 2‐year CSS was performed, demonstrating poor performance in the research (F1 score = 0.73 [95% CI 0.66–0.72], AUC = 0.66 [95% CI 0.60–0.72]) and OIS datasets (F1‐score = 0.64 [95% CI 0.56–0.72], AUC = 0.69 [95% CI 0.57–0.81]).

### 3.5 Matched research dataset model validation

The matched research dataset with 9 variables generated slightly lower but comparable metrics to the research dataset with 23 variables (Tables 4 and S4). The RF model had the best performance in the whole and larynx cohorts, with comparable performance by the SVM model in the larynx cohort. Consistent with the OIS and research datasets, prediction of 2‐year CSS in the oral cavity cohort was poor. In the oropharynx cohort, the GBT and RF models had comparably high‐performance metrics. Like the OIS dataset, stage was an influential feature in the prediction of 2‐year CSS (Figure 3).

### 3.6 Model validation by treatment modality

To allow comparison of models on homogenous populations adjusted for treatment modality, ML models were applied to the research and matched research datasets using the whole HNC cohort and separated by the treatment modalities: radiotherapy only, surgery plus adjuvant radiotherapy, and chemoradiotherapy. Due to the small sample sizes, modeling was not performed on individual HNC sites, nor on the OIS dataset due to missing surgical and chemotherapy data. The tree‐based models (GBT and RF) demonstrated the best performance for each treatment group, and compared to all treatment types, the chemoradiotherapy treated group demonstrated superior model performance, the radiotherapy only group was comparable, and the surgery plus adjuvant radiotherapy group was inferior (Table S5).

## 4 DISCUSSION

Research data sourced from a digital OIS about patients with HNC treated with radiotherapy at a major Australian metropolitan teaching and tertiary referral hospital identified more prognostic factors for 2‐year head and neck CSS compared to data collected for routine clinical practice. High‐performance ML models were generated for the whole cohort, and independently for the larynx and oropharynx cohorts using the research and OIS datasets. To the best of our knowledge, this is the first study to develop and internally validate ML models for the prediction of 2‐year CSS in patients with HNC. Our ML results demonstrate that patient, tumor, and treatment factors can be used to predict 2‐year head and neck CSS with high accuracy, with certain factors ranked more important for prediction. Our results support the use of ML for the prediction of 2‐year head and neck CSS outcomes using routinely collected clinical data from an oncology information system, with multivariable ML performance demonstrating superiority over univariable modeling. The clinical utility of these findings confirms that hospitals and clinicians may not need to implement resource intensive strategies to generate datasets for prediction modeling. With changes in treatment techniques, these models may help with determining patient suitability and preferences.

Stage is considered a strong prognostic factor in HNC. Higher stage is associated with increased risk of death at 1 year,^23^ 2 years,^24^, ^25^ 5 years (in recurrent HNC),^26^ or at any time after diagnosis^27^; however, the latter study included patients with distant metastases at presentation. Our results in a cohort without distant metastases support this. In both datasets, T3 and T4 classification was associated with increased risk of death compared to T1 classification, while T2 classification increased the risk of HNC death in the research dataset only. The association of stage in head and neck, larynx, and oropharynx cancer death is further supported as the most prominent feature in our models, with T classification being the strongest prognostic factors. Radiotherapy treatment duration (days) was also identified as an important feature in prediction. Variation in feature importance was observed in our models. For example, the OIS and restricted research datasets contained the same nine input variables; however, the importance of the variables differed despite using the same random forest classifier to score feature importance. This may be potentially explained by the differences in patient numbers and distribution of the categorical variable classes between these two datasets.

Older age and tobacco smoking have previously been shown to increase the risk of death with HNC within 2 years in one study by Thompson et al.^24^; however, this was not evident in our study, even when age was assessed as a dichotomous or continuous variable and with complete data in the research dataset. This is likely due to the statistical univariate cut‐off used in our study (*p* < 0.05) compared to Thompson et al. (*p* ≤ 0.10), meaning tobacco smoking was not included in our multivariate model. We found that tumor site, hypothyroidism, and fitness for operation predicted HNC death. CSS has been demonstrated to be higher in patients with larynx cancer compared to those with oropharynx cancer at one,^23^ and 5 years.^26^ Larynx patients also have improved 5‐year CSS when compared to oral cavity patients at 5 years.^26^ CSS at any time is higher in patients with nasopharynx cancer compared to oropharynx or hypopharynx cancers.^27^ Our results add to this body of work, whereby patients with salivary gland, oral cavity, and hypopharynx cancer have worse 2‐year CSS compared to patients with oropharynx cancer. Fitness for operation is an important patient factor which influences hypopharyngeal CSS^1^; however, our results demonstrate that this feature may also be important in other head and neck subsites, warranting further investigation. Our study demonstrated that a high proportion of patients classified as unfit (74%) and fit for surgery (98%) had good performance status (0–1). We identified an association between fitness for surgery and CSS and no association between functional capacity alone (performance status) and CSS. Despite a statistical association between hypothyroidism and CSS, clinically, there is no known relationship.

In this study, the best ML models for the prediction of 2‐year CSS in all populations and datasets were the RF and GBT models, followed by the SVM model. Deist et al. supports our findings, demonstrating that an RF model had the best performance on six out of 12 radiation oncology datasets, of which three contained patients with HNC.^28^ There are no available models predicting 2‐year head and neck CSS in patients at the site and subsite level, however, a similar study has used a model developed by Egelmeer et al.^29^ to externally validate 2‐year OS in a laryngeal carcinoma cohort using a related class of routinely collected clinical features. The authors reported a model with acceptable performance, supporting the models use in prognosis prediction.^6^ Though a direct comparison cannot be made, our best model predicted 2‐year laryngeal CSS using similar clinical features and population size with excellent performance (GBT and RF, AUC = 0.98).

With respect to CSS prediction, there is evidence of ML modeling in oral and hypopharynx cohorts. Three studies have investigated models for the prediction of 5‐year oral CSS. Tseng et al. reported a 10‐fold cross‐validation accuracy of 0.958 with a decision tree model, 0.939 with an ANN model, and 0.676 with an LR model.^12^ Campisi et al. reported a test set accuracy of 0.786, sensitivity of 0.677, specificity of 0.906, and AUC of 0.875 using an ANN model.^10^ Kim et al. reported a concordance index (C‐statistic), similar to AUC however incorporates time‐to‐event,^30^ of 0.781, 0.764, and 0.694 for the DeepSurv, RF, and CR models respectively.^11^ A subsequent study by Tseng et al. reported a concordance index of 0.689 for the prediction of 30‐month oral CSS using CR.^13^ Lastly, Tang et al. reported a concordance index of 0.764 for the prediction of CSS at any time in a cohort of patients with hypopharynx cancer using a CR model.^14^ The timing of our prediction outcome was different to these studies, however, our best models (GBT and RF) demonstrated very high AUC metric values for the whole cohort (AUC = 0.97), larynx (AUC = 0.98), and oropharynx cohorts (AUC = 0.99). Our models scored acceptable performance for the prediction of 2‐year oral cavity CSS (AUC = 0.72), much lower than Campisi et al.^10^ likely due to the smaller sample size.

There were limitations to this study. This is a single‐site investigation, and larger datasets from multiple sites should be used to investigate site‐specific HNC prognostic factors and ML prediction models. The OIS dataset contained more missing data, with fewer patients and predictors for analysis compared to the research dataset, since many user‐defined fields in the OIS were not utilized. Curation of the research dataset involved multiple researchers/data managers, where differences in data interpretation may be present. Despite these limitations, the OIS dataset still generated high‐performing models for the prediction of 2‐year head and neck, laryngeal, and oropharyngeal CSS. OIS datasets are readily available within a hospital setting, while research datasets are time and resource intensive, therefore, in the absence of research datasets, institutions should still consider using OIS datasets to inform clinical practice. Gupta et al. also supports our recommendation for the use of routinely collected OIS data to predict cancer outcomes.^31^ Therefore, to improve OIS data quality and completion, approaches such as natural language processing for automated data mining from clinical documents in the OIS should be investigated to create readily available research datasets,^32^, ^33^ or improve the entry of data in the OIS fields by implementing strategies that require completion of all data fields.^34^

## 5 CONCLUSION

Datasets extracted from an OIS for routine clinical practice and reporting, and separately for research purposes, have demonstrated utility for informing clinical practice and 2‐year CSS outcomes for patients with HNC. A comparison of multiple ML models demonstrated highest performance by the tree‐based models (GBT and RF). Data collected for research purposes identified more prognostic factors for 2‐year head and neck CSS compared to data collected for routine clinical practice and reporting. However, in the absence of research datasets, institutions can use routinely collected clinical information stored in an OIS for ML prediction of HNC survival outcomes to evaluate clinical practice and develop a better understanding of the prognostic information for each new patient.

## Supporting information


**Figure S1** Data analysis flowchart for identification of prognostic factors and prediction of 2‐year head and neck cancer‐specific survival (HNCSS). ML, machine learning; OIS, oncology information system. *Previously performed by members of the Prince of Wales Hospital Head & Neck Research group.
**Figure S2** Machine learning analysis flowchart for the prediction of 2‐year head and neck cancer‐specific survival (HNCSS).
**Table S1** Data availability and use in machine learning models.
**Table S2** Machine learning model validation performance using the research dataset (*n* = 23 variables).
**Table S3** Machine learning model validation performance using the OIS dataset (*n* = 9 variables).
**Table S4** Machine learning model validation performance using the matched research dataset (*n* = 9 variables).
**Table S5** Machine learning model validation performance by treatment modality in the whole cohort using the research and matched research dataset.

## Data Availability

Data are not available due to privacy/ethical restrictions.
