# Supplementary material for: Empirical comparison of routinely collected electronic health record data for head and neck cancer‐specific survival in machine‐learnt prognostic models
Source: Head Neck. 2022 Nov 11;45(2):365–79. doi: 10.1002/hed.27241 (PMC10100433; doi:10.1002/hed.27241)
Supplement: Supplementary file 1 — Figure S1 Data analysis flowchart for identification of prognostic factors and prediction of 2‐year head and neck cancer‐specific survival (HNCSS). ML, machine learning; OIS, oncology information system. *Previously performed by members of the Prince of Wales Hospital Head & Neck Research group. Figure S2 Machine learning analysis flowchart for the prediction of 2‐year head and neck cancer‐specific survival (HNCSS). Table S1 Data availability and use in machine learning models. Table S2 Machine learning model validation performance using the research dataset (n = 23 variables). Table S3 Machine learning model validation performance using the OIS dataset (n = 9 variables). Table S4 Machine learning model validation performance using the matched research dataset (n = 9 variables). Table S5 Machine learning model validation performance by treatment modality in the whole cohort using the research and matched research dataset. [file HED-45-365-s001.docx]

Supplement Figure 1. Data analysis flowchart for identification of prognostic factors and prediction of two-year head and neck cancer-specific survival (HNCSS). ML, machine learning; OIS, oncology information system. *Previously performed by members of the Prince of Wales Hospital Head & Neck Research group.


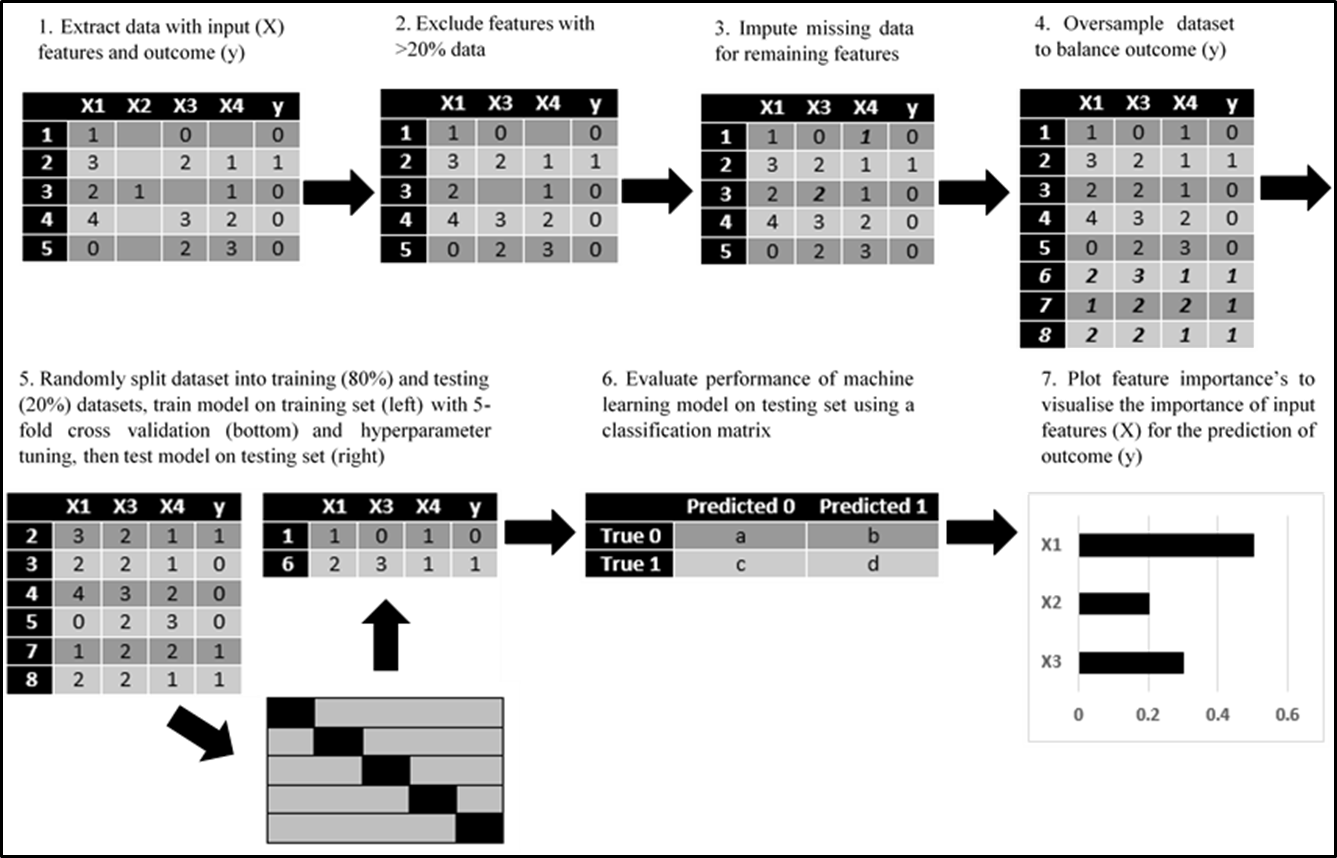


Supplement Figure 2. Machine learning analysis flowchart for the prediction of two-year head and neck cancer-specific survival (HNCSS).

| **Supplement Table 1. Data availability and use in machine learning models** | | | | | |
| --- | --- | --- | --- | --- | --- |
|  |  | **Research dataset** | | **OIS dataset** | |
| **Predictors** | **Variable name in datasets** | **Data availability** | **Used in machine learning** | **Data availability** | **Used in machine learning** |
| ***Patient*** |  |  |  |  |  |
| Age | Age | Yes | Yes | Yes | Yes |
| Gender | Gender | Yes | Yes | Yes | Yes |
| Diabetes | Diabetes | Yes | Yes | No | No |
| Hypertension | Hypertension | Yes | Yes | No | No |
| Hypothyroidism | Hypothyroidism | Yes | Yes | No | No |
| Previous HN tumour | PreviousHN | Yes | Yes | No | No |
| Previous lung tumour | PreviousLung | Yes | Yes | No | No |
| Previous other tumour | PreviousOther | Yes | Yes | No | No |
| Tobacco smoking | Tobacco | Yes | Yes | No | No |
| Alcohol consumption | Alcohol | Yes | Yes | No | No |
| Fitness for operation | FitnessOperation | Yes | Yes | No | No |
| Performance status | PerformanceScore | Yes | Yes | Yes | No |
| HPV status | HPV | No | No | No | No |
| ***Tumour*** |  |  |  |  |  |
| Tumour site | TumourSite | Yes | Yes | Yes | Yes |
| Tumour grade | Grade | Yes | Yes | Yes | No |
| Cancer operable | CancerOperable | Yes | Yes | No | No |
| T classification | Tstage | Yes | Yes | Yes | Yes |
| N classification | Nstage | Yes | Yes | Yes | Yes |
| TNM stage | OverallStage | Yes | Yes | Yes | Yes |
| ***Treatment*** |  |  |  |  |  |
| Radiotherapy dose (Gy) | Dose | Yes | Yes | Yes | Yes |
| Radiotherapy fractions | Fractions | Yes | Yes | Yes | Yes |
| Radiotherapy duration (days) | Days | Yes | Yes | Yes | Yes |
| Chemotherapy | Chemotherapy | Yes | Yes | Yes | No |
| Surgery | Surgery | Yes | Yes | No | No |
| **TOTAL** | **24** | **23** | **23** | **12** | **9** |

Gy, gray; HN, head and neck; HPV, human papillomavirus; OIS, oncology information system

| **Supplement Table 2. Machine learning model validation performance using the research dataset (n=23 variables)** | | | | | | |
| --- | --- | --- | --- | --- | --- | --- |
|  | | **Model performance metrics (95% CI)** | | | | |
| **Site** | Metric | Logistic regression | Gradient boosted trees | Random forest | Support vector machine | Artificial neural network |
| Whole cohort | Recall  Specificity  Precision  F1-score  AUC | 0.91 (0.85-0.97)  0.54 (0.44-0.64)  0.66 (0.58-0.74)  0.77 (0.71-0.83)  0.76 (0.71-0.81) | **0.86 (0.79-0.93)**  **0.93 (0.88-0.98)**  **0.93 (0.87-0.99)**  **0.89 (0.84-0.94)**  **0.96 (0.94-0.98)** | **0.85 (0.78-0.92)**  **0.94 (0.89-0.99)**  **0.94 (0.89-0.99)**  **0.89 (0.84-0.94)**  **0.97 (0.95-0.99)** | 0.95 (0.90-1.00)  0.73 (0.64-0.82)  0.78 (0.70-0.86)  0.86 (0.81-0.91)  0.84 (0.80-0.88) | 0.81 (0.73-0.89)  0.57 (0.47-0.67)  0.65 (0.56-0.74)  0.72 (0.66-0.78)  0.76 (0.71-0.81) |
| Larynx | Recall  Specificity  Precision  F1-score  AUC | 1.00 (1.00-1.00)  0.82 (0.68-0.96)  0.85 (0.73-0.97)  0.92 (0.85-0.99)  0.92 (0.87-0.97) | **0.96 (0.89-1.00)**  **0.89 (0.77-1.00)**  **0.90 (0.79-1.00)**  **0.93 (0.86-1.00)**  **0.98 (0.95-1.00)** | 0.96 (0.89-1.00)  0.82 (0.68-0.96)  0.84 (0.71-0.97)  0.90 (0.82-0.98)  0.98 (0.95-1.00) | 1.00 (1.00-1.00)  0.86 (0.73-0.99)  0.88 (0.77-0.99)  0.93 (0.87-0.99)  0.93 (0.88-0.98) | 1.00 (1.00-1.00)  0.46 (0.28-0.64)  0.65 (0.51-0.79)  0.79 (0.70-0.88)  0.82 (0.74-0.90) |
| Oral cavity | Recall  Specificity  Precision  F1-score  AUC | 0.77 (0.54-1.00)  0.31 (0.06-0.56)  0.53 (0.31-0.75)  0.62 (0.45-0.79)  0.52 (0.36-0.68) | 0.62 (0.36-0.88)  0.54 (0.27-0.81)  0.57 (0.31-0.83)  0.59 (0.40-0.78)  0.64 (0.49-0.79) | **0.62 (0.36-0.88)**  **0.69 (0.44-0.94)**  **0.67 (0.40-0.94)**  **0.64 (0.45-0.83)**  **0.69 (0.55-0.83)** | 0.92 (0.77-1.00)  0.38 (0.12-0.64)  0.60 (0.39-0.81)  0.73 (0.58-0.88)  0.40 (0.25-0.55) | 0.85 (0.66-1.00)  0.08 (0-0.23)  0.48 (0.28-0.68)  0.61 (0.45-0.77)  0.43 (0.27-0.59) |
| Oropharynx | Recall  Specificity  Precision  F1-score  AUC | 0.92 (0.83-1.00)  0.73 (0.59-0.87)  0.77 (0.64-0.90)  0.84 (0.76-0.92)  0.82 (0.75-0.89) | 0.86 (0.75-0.97)  0.92 (0.83-1.00)  0.91 (0.81-1.00)  0.89 (0.82-0.96)  0.98 (0.96-1.00) | **0.92 (0.83-1.00)**  **0.97 (0.92-1.00)**  **0.97 (0.91-1.00)**  **0.94 (0.88-1.00)**  **0.99 (0.97-1.00)** | 1.00 (1.00-1.00)  0.76 (0.62-0.90)  0.80 (0.68-0.92)  0.89 (0.82-0.96)  0.92 (0.87-0.97) | 0.67 (0.52-0.82)  0.86 (0.75-0.97)  0.83 (0.69-0.97)  0.74 (0.63-0.85)  0.81 (0.74-0.88) |

95% CI, 95% confidence interval; AUC, area under curve

Bold indicates best overall performing model

| **Supplement Table 3. Machine learning model validation performance using the OIS dataset (n=9 variables)** | | | | | | |
| --- | --- | --- | --- | --- | --- | --- |
|  | | **Model performance metrics (95% CI)** | | | | |
| **Site** | Metric | Logistic regression | Gradient boosted trees | Random forest | Support vector machine | Artificial neural network |
| Whole cohort | Recall  Specificity  Precision  F1-score  AUC | 0.77 (0.67-0.87)  0.63 (0.52-0.74)  0.67 (0.57-0.77)  0.72 (0.65-0.79)  0.74 (0.68-0.80) | 0.78 (0.68-0.88)  0.95 (0.90-1.00)  0.93 (0.87-0.99)  0.85 (0.79-0.91)  0.94 (0.91-0.97) | **0.85 (0.77-0.93)**  **0.96 (0.92-1.00)**  **0.95 (0.90-1.00)**  **0.90 (0.85-0.95)**  **0.94 (0.91-0.97)** | 0.79 (0.70-0.88)  0.79 (0.70-0.88)  0.79 (0.70-0.88)  0.79 (0.73-0.86)  0.88 (0.84-0.92) | 0.42 (0.31-0.53)  0.70 (0.59-0.81)  0.58 (0.45-0.71)  0.49 (0.40-0.58)  0.66 (0.60-0.72) |
| Larynx | Recall  Specificity  Precision  F1-score  AUC | 0.96 (0.88-1.00)  0.75 (0.58-0.92)  0.79 (0.64-0.94)  0.87 (0.78-0.96)  0.93 (0.88-0.98) | 0.88 (0.75-1.00)  0.92 (0.81-1.00)  0.91 (0.79-1.00)  0.89 (0.80-0.98)  0.98 (0.95-1.00) | **0.88 (0.75-1.00)**  **0.96 (0.88-1.00)**  **0.95 (0.86-1.00)**  **0.91 (0.83-0.99)**  **0.98 (0.95-1.00)** | **1.00 (1.00-1.00)**  **0.92 (0.81-1.00)**  **0.92 (0.82-1.00)**  **0.96 (0.91-1.00)**  **0.95 (0.90-1.00)** | 1.00 (1.00-1.00)  0.63 (0.44-0.82)  0.73 (0.58-0.88)  0.84 (0.74-0.94)  0.84 (0.76-0.92) |
| Oral cavity | Recall  Specificity  Precision  F1-score  AUC | 0.67 (0.40-0.94)  0.38 (0.12-0.64)  0.50 (0.26-0.75)  0.57 (0.39-0.75)  0.57 (0.41-0.73) | **0.67 (0.40-0.94)**  **0.69 (0.44-0.94)**  **0.67 (0.40-0.94)**  **0.67 (0.48-0.86)**  **0.72 (0.58-0.86)** | 0.50 (0.22-0.78)  0.69 (0.44-0.94)  0.60 (0.30-0.90)  0.55 (0.34-0.76)  0.60 (0.44-0.76) | 0.50 (0.22-0.78)  0.54 (0.27-0.81)  0.50 (0.22-0.78)  0.50 (0.30-0.70)  0.53 (0.37-0.69) | 0.75 (0.51-1.00)  0.31 (0.06-0.56)  0.50 (0.27-0.73)  0.60 (0.42-0.78)  0.52 (0.36-0.68) |
| Oropharynx | Recall  Specificity  Precision  F1-score  AUC | 0.82 (0.68-0.96)  0.79 (0.64-0.94)  0.79 (0.64-0.94)  0.81 (0.71-0.91)  0.90 (0.84-0.96) | **0.82 (0.68-0.96)**  **1.00 (1.00-1.00)**  **1.00 (1.00-1.00)**  **0.90 (0.82-0.98)**  **0.99 (0.97-1.00)** | **0.89 (0.77-1.00)**  **0.96 (0.89-1.00)**  **0.96 (0.88-1.00)**  **0.93 (0.86-1.00)**  **0.98 (0.95-1.00)** | 0.96 (0.89-1.00)  0.68 (0.51-0.85)  0.75 (0.61-0.89)  0.84 (0.75-0.93)  0.86 (0.79-0.93) | 0.46 (0.28-0.64)  1.00 (1.00-1.00)  1.00 (1.00-1.00)  0.63 (0.48-0.78)  0.93 (0.88-0.98) |

95% CI, 95% confidence interval; AUC, area under curve; OIS, oncology information system

Bold indicates best overall performing model

| **Supplement Table 4. Machine learning model validation performance using the matched research dataset (n=9 variables)** | | | | | | |
| --- | --- | --- | --- | --- | --- | --- |
|  | | **Model performance metrics (95% CI)** | | | | |
| **Site** | Metric | Logistic regression | Gradient boosted trees | Random forest | Support vector machine | Artificial neural network |
| Whole cohort | Recall  Specificity  Precision  F1-score  AUC | 0.80 (0.72-0.88)  0.53 (0.43-0.63)  0.62 (0.53-0.71)  0.70 (0.64-0.76)  0.71 (0.66-0.76) | 0.88 (0.81-0.95)  0.89 (0.82-0.96)  0.89 (0.82-0.96)  0.88 (0.83-0.93)  0.95 (0.93-0.97) | **0.88 (0.81-0.95)**  **0.90 (0.84-0.96)**  **0.90 (0.84-0.96)**  **0.89 (0.84-0.94)**  **0.95 (0.93-0.97)** | 0.69 (0.59-0.79)  0.93 (0.88-0.98)  0.91 (0.84-0.98)  0.79 (0.73-0.85)  0.93 (0.90-0.96) | 0.64 (0.54-0.74)  0.63 (0.53-0.73)  0.63 (0.53-0.73)  0.64 (0.57-0.71)  0.70 (0.65-0.75) |
| Larynx | Recall  Specificity  Precision  F1-score  AUC | 1.00 (1.00-1.00)  0.75 (0.59-0.91)  0.80 (0.67-0.93)  0.89 (0.81-0.97)  0.84 (0.77-0.91) | 0.89 (0.77-1.00)  0.86 (0.73-0.99)  0.86 (0.73-0.99)  0.88 (0.80-0.96)  0.96 (0.92-1.00) | **0.96 (0.89-1.00)**  **0.86 (0.73-0.99)**  **0.87 (0.75-0.99)**  **0.92 (0.85-0.99)**  **0.97 (0.94-1.00)** | **1.00 (1.00-1.00)**  **0.86 (0.73-0.99)**  **0.88 (0.77-0.99)**  **0.93 (0.87-0.99)**  **0.94 (0.89-0.99)** | 0.96 (0.89-1.00)  0.75 (0.59-0.91)  0.79 (0.65-0.93)  0.87 (0.79-0.95)  0.82 (0.74-0.90) |
| Oral cavity | Recall  Specificity  Precision  F1-score  AUC | 0.92 (0.77-1.00)  0.23 (0-0.46)  0.55 (0.34-0.76)  0.69 (0.54-0.84)  0.56 (0.40-0.72) | 0.69 (0.44-0.94)  0.38 (0.12-0.64)  0.53 (0.29-0.77)  0.60 (0.42-0.78)  0.66 (0.51-0.81) | 0.69 (0.44-0.94)  0.54 (0.27-0.81)  0.60 (0.35-0.85)  0.64 (0.46-0.82)  0.73 (0.59-0.87) | **0.77 (0.54-1.00)**  **0.62 (0.36-0.88)**  **0.67 (0.43-0.91)**  **0.71 (0.54-0.88)**  **0.67 (0.52-0.82)** | 1.00 (1.00-1.00)  0  0.50 (0.31-0.69)  0.67 (0.52-0.82)  0.36 (0.21-0.51) |
| Oropharynx | Recall  Specificity  Precision  F1-score  AUC | 0.75 (0.61-0.89)  0.65 (0.50-0.80)  0.68 (0.54-0.82)  0.71 (0.61-0.81)  0.73 (0.65-0.81) | **0.89 (0.79-0.99)**  **0.92 (0.83-1.00)**  **0.91 (0.82-1.00)**  **0.90 (0.83-0.97)**  **0.93 (0.87-0.97)** | 0.86 (0.75-0.97)  0.92 (0.83-1.00)  0.91 (0.81-1.00)  0.89 (0.82-0.96)  0.96 (0.93-0.99) | 0.86 (0.75-0.97)  0.65 (0.50-0.80)  0.70 (0.56-0.84)  0.78 (0.69-0.87)  0.80 (0.73-0.87) | 0.72 (0.57-0.87)  0.57 (0.41-0.73)  0.62 (0.47-0.77)  0.67 (0.57-0.77)  0.74 (0.66-0.82) |

95% CI, 95% confidence interval; AUC, area under curve

Bold indicates best overall performing model

| **Supplement Table 5. Machine learning model validation performance by treatment modality in the whole cohort using the research and matched research dataset** | | | | | |
| --- | --- | --- | --- | --- | --- |
|  | | **Model performance metrics (95% CI)** | | | |
|  |  | **Research dataset** | | **Matched research dataset** | |
|  |  | Gradient boosted trees | Random forest | Gradient boosted trees | Random forest |
| All treatment types | Recall  Specificity  Precision  F1-score  AUC | 0.86 (0.79-0.93)  0.93 (0.88-0.98)  0.93 (0.87-0.99)  0.89 (0.84-0.94)  0.96 (0.94-0.98) | 0.85 (0.78-0.92)  0.94 (0.89-0.99)  0.94 (0.89-0.99)  0.89 (0.84-0.94)  0.97 (0.95-0.99) | 0.86 (0.79-0.93)  0.93 (0.88-0.98)  0.93 (0.87-0.99)  0.89 (0.84-0.94)  0.96 (0.94-0.98) | 0.85 (0.78-0.92)  0.94 (0.89-0.99)  0.94 (0.89-0.99)  0.89 (0.84-0.94)  0.97 (0.95-0.99) |
| Radiotherapy only | Recall  Specificity  Precision  F1-score  AUC | 0.88 (0.82-0.94)  0.88 (0.80-0.97)  0.88 (0.77-0.99)  0.88 (0.77-0.99)  0.96 (0.89-1.00) | 0.91 (0.86-0.96)  0.88 (0.80-0.96)  0.88 (0.77-0.99)  0.89 (0.78-1.00)  0.97 (0.91-1.00) | 0.84 (0.77-0.91)  0.85 (0.76-0.94)  0.84 (0.71-0.97)  0.84 (0.77-0.91)  0.96 (0.89-1.00) | 0.88 (0.82-0.94)  0.82 (0.73-0.91)  0.82 (0.69-0.95)  0.85 (0.73-0.97)  0.94 (0.86-1.00) |
| Surgery plus adjuvant radiotherapy | Recall  Specificity  Precision  F1-score  AUC | 0.79 (0.71-0.87)  0.86 (0.77-0.95)  0.85 (0.72-0.98)  0.81 (0.66-0.96)  0.89 (0.77-1.00) | 0.75 (0.66-0.84)  1.00 (1.00-1.00)  1.00 (1.00-1.00)  0.86 (0.73-0.99)  0.91 (0.79-1.00) | 0.75 (0.66-0.84)  0.93 (0.86-1.00)  0.91 (0.80-1.00)  0.82 (0.68-0.96)  0.89 (0.76-1.00) | 0.75 (0.66-0.84)  0.93 (0.86-1.00)  0.91 (0.80-1.00)  0.82 (0.68-0.96)  0.89 (0.76-1.00) |
| Chemoradiotherapy | Recall  Specificity  Precision  F1-score  AUC | 0.91 (0.85-0.97)  0.96 (0.90-1.00)  0.95 (0.86-1.00)  0.93 (0.83-1.00)  0.98 (0.92-1.00) | 1.00 (1.00-1.00)  0.96 (0.90-1.00)  0.96 (0.88-1.00)  0.98 (0.92-1.00)  0.99 (0.95-1.00) | 0.83 (0.75-0.92)  0.87 (0.77-0.97)  0.86 (0.72-1.00)  0.84 (0.69-0.99)  0.94 (0.84-1.00) | 0.87 (0.80-0.94)  0.78 (0.66-0.90)  0.80 (0.64-0.96)  0.83 (0.68-0.98)  0.94 (0.85-1.00) |

95% CI, 95% confidence interval; AUC, area under curve
